# Therapeutic Effects of Human Mesenchymal Stem Cells in Wistar-Kyoto Rats with Anti-Glomerular Basement Membrane Glomerulonephritis

**DOI:** 10.1371/journal.pone.0067475

**Published:** 2013-06-24

**Authors:** Taihei Suzuki, Masayuki Iyoda, Takanori Shibata, Hirokazu Ohtaki, Kei Matsumoto, Yuki Shindo-Hirai, Yoshihiro Kuno, Yukihiro Wada, Yasutaka Yamamoto, Mio Kawaguchi, Seiji Shioda, Tadao Akizawa

**Affiliations:** 1 Division of Nephrology, Department of Medicine, Showa University School of Medicine, Tokyo, Japan; 2 Department of Anatomy, Showa University School of Medicine, Tokyo, Japan; 3 Department of Respiratory Medicine, Institute of Clinical Medicine, University of Tsukuba, Tsukuba, Japan; Institut National de la Santé et de la Recherche Médicale, France

## Abstract

**Introduction:**

Multipotent mesenchymal stem cells (MSCs) have become a promising therapeutic approach in many clinical conditions. The hypothesis that MSCs can provide a potential therapy for human anti-glomerular basement membrane (GBM) glomerulonephritis (GN) was tested.

**Methods:**

Nephrotoxic serum nephritis was induced in Wistar-Kyoto rats on day 0. Groups of animals were given either human MSCs (hMSCs, 3×10^6^) or vehicle by intravenous injection on day 4; all rats were sacrificed at either day 7 or day 13.

**Results:**

Fluorescently labeled hMSCs were localized in glomeruli and tubulointerstitium 5 h after hMSC administration and persisted until 48 h, but hMSCs were barely detectable after 7 days. hMSC-treated rats had decreased kidney weight, proteinuria, and glomerular tuft area at each time point. The serum creatinine level and degree of glomerular crescent formation were decreased by hMSC treatment on day 13. ED1-positive macrophages, CD8-positive cells, and TUNEL-positive apoptotic cells in glomeruli were reduced by hMSC treatment on day 7, and this trend in apoptotic cells persisted to day 13. Renal cortical mRNA for TNF-α, IL-1β, and IL-17, and the serum IL-17A level were decreased, whereas renal cortical mRNA for IL-4 and Foxp3 and the serum IL-10 level were increased in the MSC-treated group on day 7. Collagen types I and III and TGF-β mRNA were decreased by hMSC treatment on day 13.

**Conclusion:**

The present results demonstrated that anti-inflammatory and immunomodulatory effects were involved in the mechanism of attenuating established experimental anti-GBM GN by hMSCs. These results suggest that hMSCs are a promising therapeutic candidate for the treatment of anti-GBM GN.

## Introduction

Stem/progenitor cells from bone marrow, referred to as mesenchymal stem cells or multipotent mesenchymal stromal cells (MSCs), are a heterogeneous population of fibroblast-like stromal cells that have been isolated from bone marrow, fat tissue, and umbilical cord. They have multipotent and self-renewing properties and can differentiate into cells of mesenchymal and nonmesenchymal origin [1], [2]. The potential of bone marrow-derived MSCs for renal repair has been shown in rodent models of lupus nephritis [3], diabetic nephropathy [4], and acute kidney injury (AKI) induced by glycerol [5], cisplatin [6], [7], [8], or ischemia-reperfusion [9], [10], [11], and in rat anti-Thy1.1 glomerulonephritis (GN) [12], [13] and obstruction-induced renal fibrosis [14]. Following their administration *in vivo*, MSCs migrate to damaged kidney tissue [5]–[7], [9], [11]–[14] where they produce an array of anti-inflammatory cytokines/chemokines that can alter the course of injury. Recent reports suggest that the benefit of MSCs is unlikely to be due to transdifferentiation into renal tubular cells [8], [9], [11], [12]. MSCs are thought to elicit repair through paracrine mechanisms that modulate the anti-inflammatory [8], [9], anti-apoptotic [7], [8], proliferative [6], [7], [8], [12], angiogenic [11], immunomodulatory [3], and anti-oxidant [10] effects resulting in tissue repair and cellular replacement. Recently, Prockop et al. reported that a multipotent anti-inflammatory protein referred to as TNF-α stimulated gene/protein 6 (TSG-6) secreted by activated MSCs was a crucial factor in attenuating myocardial infarction and zymosan-induced peritonitis in mice [15], [16]. On the basis of these promising experimental studies, clinical trials of the use of human MSCs (hMSCs) in patients with kidney transplantation, AKI following cisplatin administration or cardiac surgery, and lupus nephritis are currently underway (data from ClinicalTrials.gov). The enlargement of regulatory T cells (Tregs) [3], [17] and restriction of memory CD8+ T cells [17] were clinically identified as important mechanisms of renal repair by MSCs in patients with kidney transplantation or lupus nephritis.

Experimental anti-glomerular basement membrane (GBM) GN is a model of rapidly progressive GN, and it is characterized by proteinuria, renal dysfunction, glomerular crescent and necrosis formation, and fibrin deposition, resembling human crescentic GN. It is now widely believed that experimental anti-GBM GN is a predominantly Th1- and Th17-dependent disease [18], [19]. Murine anti-GBM GN was reduced by administration of Th2 cytokines [20] and endogenous Tregs [21] and deteriorated by inducing GN in Treg-depleted mice [22]. In this study, the therapeutic effects of human bone marrow-derived MSCs and their mechanism in Wistar-Kyoto (WKY) rats with anti-GBM GN were investigated. The hypo-immunogenic character of human bone marrow-derived MSCs, which do not express human leukocyte antigen (HLA) major histocompatibility complex (MHC) class II and co-stimulatory molecules, allows their use in xenotransplantation studies without an obvious immune response [14], [23], [24], [25]. A recent study presented additional evidence for the hypoimmunogenic property of hMSCs implanted into the rat striatum [26].

In the clinical setting, GN patients already have renal dysfunction and/or proteinuria when treatment starts. Therefore, hMSCs were administered to rats 4 days after the induction of nephritis, that is, after the rats had already developed established GN. Treatment with hMSCs significantly attenuated the progression of anti-GBM GN by anti-inflammatory and immunomodulatory effects, even after the establishment of nephritis that, in turn, blunted subsequent development of glomerular fibrosis.

## Materials and Methods

### Preparation of cells

Frozen vials of hMSCs from bone marrow were obtained from the Center for the Preparation and Distribution of Adult Stem Cells (http://medicine.tamhsc.edu/irm/msc-distribution.html) that supplies standardized preparations of MSCs enriched for early progenitor cells to > 300 laboratories under the auspices of a National Institutes of Health (NIH)/National Center for Research Resources grant (P40 RR 17 447-06). The experiments were performed with hMSCs from donor 281L. To expand hMSCs, a frozen vial of 1.0×10^6^ passage 3 cells was thawed and plated at 80 cells/cm^2^ in multiple 180 cm^2^ plates with 30 mL complete culture medium (CCM) that consisted of a-minimal essential medium (α-MEM; Invitrogen), 20% FBS, 100 units/mL penicillin, 100 mg/mL streptomycin (Invitrogen), and 2 mM L-glutamine (Invitrogen). The cultures were incubated, and the medium was replaced every 3 days for approximately 7 days until they were 70–80% confluent. The medium was discarded, the cultures were washed with PBS, adherent cells were harvested with 0.25% trypsin and 1 mM EDTA for 5 minutes at 37°C, and the cells were resuspended at 3.0×10^6^ cells in 1000 µL of sterile HBSS for injection.

### Ethics statement

All animals were given humane care in compliance with institutional guidelines using protocols approved by the Animal Care Committee of Showa University in Tokyo.

### Experimental protocol

Seven-week-old, female WKY rats weighing 135–150 g were purchased from Charles River Japan (Atsugi, Kanagawa, Japan) and used in all of the experiments. The animals were housed in the animal care facility of Showa University (25°C, 50% humidity, 12-hour dark/light cycle) with free access to food and water. A total of 45 female WKY rats was injected intravenously with 20 µl of nephrotoxic serum, which was prepared as described previously [27], on day 0. Groups of animals were given either hMSCs (3×10^6^) or vehicle by intravenous injection on day 4; all rats were sacrificed on either day 7 or day 13. Vehicle-treated groups received an equal volume of Hank’s balanced salt solution (HBSS). For each group, 10 to 14 rats were analyzed. Six female WKY rats at the age of 7 weeks were used as normal controls. At the end of the study, the rats were anesthetized, their blood was collected by cardiac puncture, and their organs were collected. Renal tissue was divided; some portions were snap-frozen in liquid nitrogen, and some portions were fixed in 2% paraformaldehyde/PBS for later use.

### Proteinuria and creatinine determination

For the analysis of proteinuria, rats were housed individually in metabolic cages for 24-hour urine collection. Urine samples were collected on the day before sacrifice. Urinary protein was determined using the Biuret method. Serum and urinary creatinine (Cr) levels were measured using an automated analyzer (Hitachi Corp., Tokyo, Japan) according to the manufacturer’s instructions.

### Measurement of urinary total collagen levels

Urinary total collagen levels were determined by analysis of urinary hydroxyproline content as described by Kivirikko et al [28]. Hydroxyproline values were converted to collagen content by multiplying by a factor of 6.94 (since hydroxyproline represents approximately 14.4% of the amino acid composition of collagen) [29] and expressed further as a proportion of the urinary Cr levels (µmol of collagen levels/mg of urinary Cr levels).

### Measurement of circulating anti-rabbit IgG antibody

The level of circulating anti-rabbit IgG antibody in rats with nephritis was measured by enzyme-linked immunosorbent assay (ELISA) according to the ELISA procedure as described previously [27], [30].

### Light microscopic study

Tissues fixed in 2% paraformaldehyde/PBS were embedded in paraffin using routine protocols. Paraffin-embedded materials were sectioned at 2 µm for routine staining with periodic-acid Schiff (PAS). One-µm-thick sections were used for periodic-acid methenamine silver stains (silver). The glomerular tuft area was quantified in 50 full-sized glomeruli (PAS stain) using WinROOF image processing software (Mitani Corp., Tokyo, Japan). The number of crescentic or necrotizing glomeruli per 100 glomeruli of each rat was calculated and expressed as a percentage. The percentage of area occupied by crescents in each glomeruli was estimated and assigned one of the following scores: 0, absent; 1, less than 1/4; 2, between 1/4 and 1/2; 3, between 1/2 and 3/4; and 4, more than 3/4 of the whole glomerulus [30]. The mean score of 50 glomeruli was then calculated as the crescent score. All histological analyses were performed without knowledge of the origin of the slides.

### Immunohistochemistry

The antibodies used in this study were: mouse anti-rat ED1 antibody (BMA, Augst, Switzerland) as a macrophage marker, mouse anti-rat CD8 antibody (Clone number: X8, Antigenix America, Huntington Station, NY, USA), mouse anti-rat endothelial cell antibody (RECA-1) (Clone number: HIS52, Serotec, Oxford, UK) as an endothelial cell surface marker, rabbit anti-human podocin antibody (Immuno-Biological Laboratories, Gunma, Japan), and goat anti-rat IL-1β (Santa Cruz Biotechnology, Santa Cruz, CA, USA). Biotinylated rabbit anti-mouse IgG or anti-goat IgG antibody and peroxidase-conjugated streptavidin (LSAB 2 kit/HRP) were purchased from Dako (Glostrup, Denmark). Immunohistochemical staining for ED1 (1∶50 dilution) and CD8 (1∶50 dilution) and two-color immunostaining with ED1 (1∶50 dilution)/IL-1β (1∶5 dilution) and RECA-1 (1∶10 dilution)/IL-1β (1∶5 dilution) were performed as described previously [30]. Cells undergoing apoptosis were identified by in situ TdT-mediated dUTP nick end labeling (TUNEL) using the ApopTag plus peroxidase in situ apoptosis detection kit (Chemicon International Inc., Temecula, CA, USA). Two-color immunostaining was used to detect colocalization of ED1 or podocin (1∶50 dilution) with TUNEL. Sections were microwaved twice for 5 min for the two-color immunostaining with ED1/TUNEL and podocin/TUNEL. CD8+ cells and TUNEL+ cells were estimated by counting the numbers of these cells within 50 glomeruli and dividing the total number by 50. The extent of ED1 staining of each glomerulus was graded for 50 glomeruli on a 4-point scale: 0, absent; 1, weak; 2, moderate; and 3, severe [30]. The mean score was then calculated as the ED1 score. All histological analyses were performed without knowledge of the origin of the slides.

### Immunofluorescence

The tissues were snap-frozen in liquid nitrogen and cut into 4-µm-thick sections. The deposits of rabbit IgG, rat C3, rat fibrin, and rat IgG in the kidney sections were evaluated using a previously described method [30]. The extent of fibrin deposition in each glomerulus was graded for 50 glomeruli using a 4-point scale: 0, absent; 1, less than 1/3; 2, between 1/3 and 2/3; and 3, more than 2/3 of the whole glomerulus. The mean score of 50 glomeruli was then calculated as the fibrin score.

### Real-time reverse transcriptase polymerase chain reaction (RT-PCR)

Gene expressions of rat TNF-α, IL-1β, IL-6, MCP-1, IFN-γ, IL-4, IL-10, IL-17, Foxp3, collagen type I, collagen type III, TGF-β, nephrin, plasminogen activator inhibitor-1 (PAI-1), platelet-derived growth factor-B (PDGF-B), and glyceraldehyde-3-phosphate dehydrogenase (GAPDH) were analyzed using real-time RT-PCR in kidney tissues (cortex) as described previously [30], [31], [32]. mRNA expressions were normalized using GAPDH as an endogenous control to correct for the differences in the amount of total RNA added to each reaction.

### Quantification of serum cytokine levels

Serum IL-17 (eBioscience, San Diego, CA, USA), IFN-γ (R&D Systems, Minneapolis, MN, USA), IL-10 (R&D Systems), IL-6 (R&D Systems), and IL-1β (R&D Systems) protein levels were determined using commercially available ELISA kits according to each manufacturer’s instructions.

### Flow cytometry

Isolated lymphocytes from the spleen were labeled with specific monoclonal antibodies (CD4-APC, CD25-FITC, and Foxp3-PE; BD PharMingen, San Diego, CA, USA). The proportion of cells positive for these molecules was determined by flow cytometry (FACScan, BD PharMingen), and the results were analyzed using Cell Quest software.

### Detection of CM-DiI-labeled hMSCs in kidneys

Twelve rats with nephritis and two rats without nephritis were administered hMSCs that were fluorescently labeled using conjugated red fluorochrome CellTracker CM-DiI for identification in histopathological sections (Invitrogen, Carlsbad, CA, USA). The presence of CM-DiI-labeled hMSCs in kidneys, lungs, liver, heart, and spleen was examined by counterstaining with actin filaments using conjugated green fluorochrome Acti-stain 488 Fluorescent Phalloidin (Cytoskeleton Inc., Denver, CO, USA). The frequency of hMSCs in each organ was graded on a 4-point scale: 0, absent; 1, faint; 2, mild; and 3, moderate.

### Statistical analysis

Data were recorded as means ± SEM. The Mann-Whitney test was performed, and values of *P*<0.05 were considered significant.

## Results

### Effects of hMSCs on biochemical parameters in rats with nephritis

**Figure 1:**
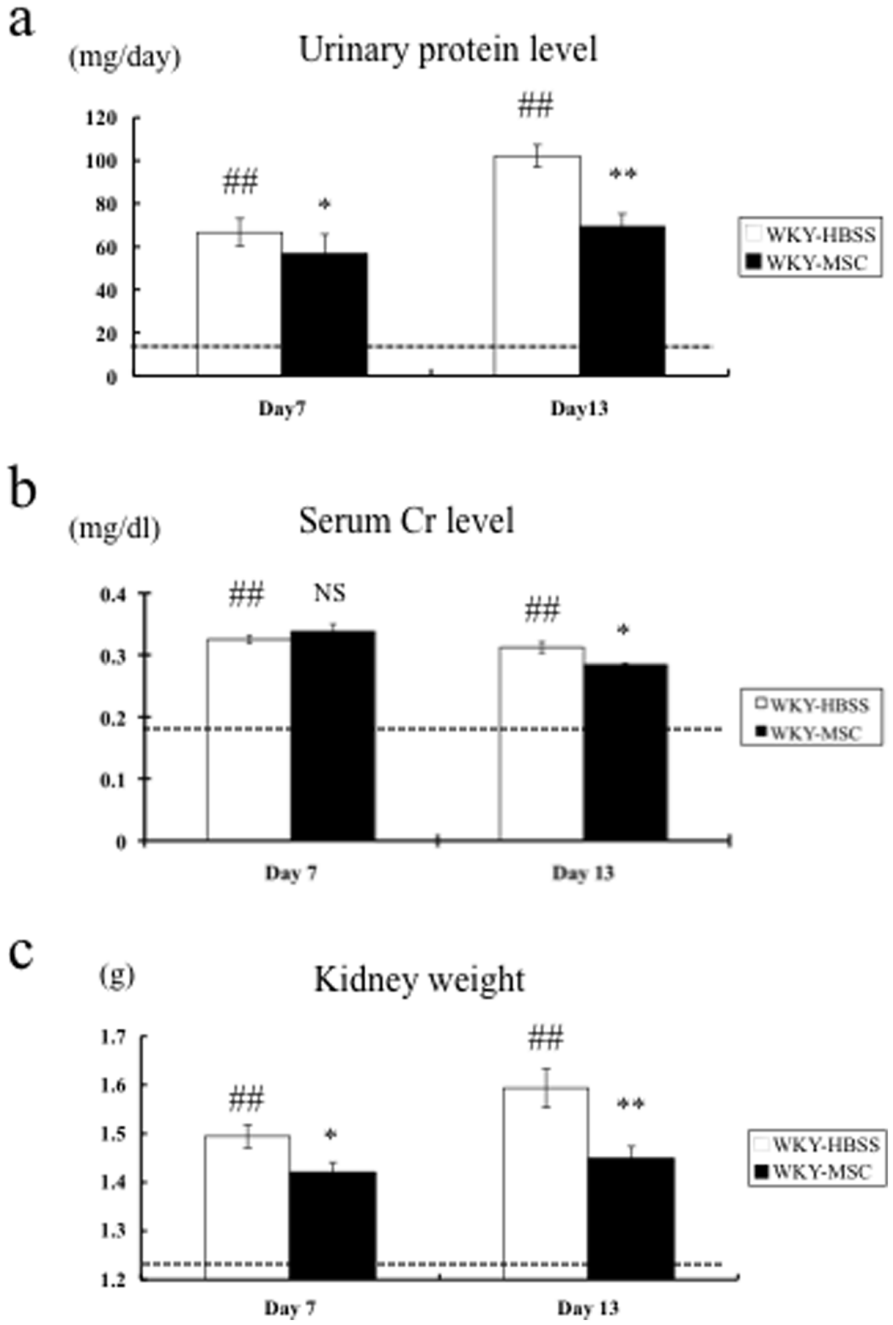
Urinary total protein level, serum creatinine level, and total kidney weight in the study groups. (**a**) Urinary total protein level, (**b**) serum creatinine level, and (**c**) total kidney weight of WKY rats with nephritis treated with either HBSS (WKY-HBSS) or MSC (WKY-MSC) are shown. The horizontal dotted lines show the level of proteinuria, serum creatinine, and kidney weight in WKY rats without nephritis (WKY-NTS (-)). Data are means ± SEM. NS, *p<0.05, **p<0.01: HBSS-treated rats with nephritis vs. MSC-treated rat with nephritis; ##p<0.01: HBSS-treated rats with nephritis vs. WKY rats without nephritis. NS, not significant.

Nephrotoxic serum nephritis was induced in WKY rats on day 0. Groups of animals were given either hMSCs or vehicle on day 4; all rats were sacrificed at either day 7 or day 13. Vehicle-treated groups received an equal volume of HBSS. There were no significant changes in body weight (BW) between the vehicle-treated control (WKY-HBSS) and hMSC-treated (WKY-MSC) rats throughout the experimental period (data not shown). **Figures 1a-1c** show the results for urinary total protein levels, serum creatinine (Cr) levels, and total kidney weight for each group. These three parameters were significantly higher in the WKY-HBSS rats than in the WKY rats without nephritis (WKY-NTS (-)) on day 7 and day 13. Urinary protein was identical between the WKY-HBSS rats and the WKY-MSC rats on day 4 when treatment started (41.71±6.26 vs. 43.67±8.88 mg/day, NS). Urinary protein in the WKY-HBSS rats reached 66.66±5.22 mg/day on day 7 and 102.10±7.52 mg/day on day 13. hMSC treatment, which was administered intravenously on day 4, significantly improved the urinary protein level to –15% and –33% on days 7 and 13, respectively (Day 7: 66.66±5.22 vs. 56.81±5.87 mg/day, p<0.05; Day 13: 102.10±7.52 vs. 69.37±5.95 mg/day, p<0.01) (**Fig.1a**). While the serum Cr level on day 7 was not different between the groups, it was decreased significantly more in the WKY-MSC rats than in the WKY-HBSS rats on day 13 (Day 7: 0.33±0.001 vs. 0.34±0.01 mg/dL, NS; Day 13: 0.31±0.001 vs. 0.28±0.01 mg/dL, p<0.05). (**Fig.1b**). hMSC treatment also suppressed kidney hypertrophy of rats with nephritis to –5% and –9% on days 7 and 13, respectively (Day 7: 1.50±0.02 vs. 1.42±0.02 g, p<0.05; Day 13: 1.59±0.04 vs. 1.45±0.02 g, p<0.01) (**Fig.1c**).

### Effects of hMSCs on renal histological findings in rats with nephritis

**Figure 2:**
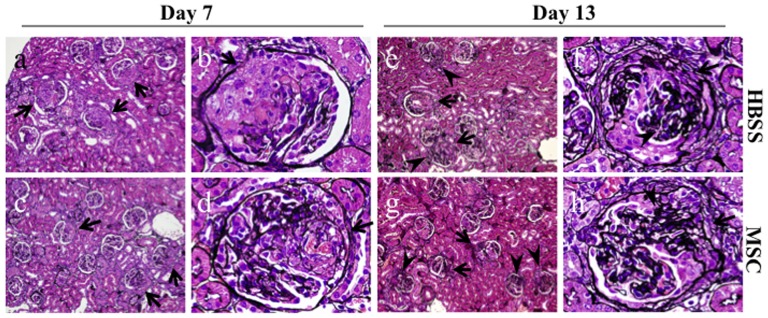
Light microscopic findings in the study groups. Representative pictures stained with silver on day 7 (**a**–**d**) and day 13 (**e**–**h**) in an HBSS-treated rat with nephritis (**a, b, e, f**) and an MSC-treated rat with nephritis (**c, d, g, h**). The glomeruli on day 7 show severe fibrinoid necrosis and cellular crescent formation (arrows) (**a, b**). The crescentic glomeruli have started to transform from cellular to fibrocellular (arrows), and there is some segmental glomerulosclerosis (arrowheads) on day 13 (**e, f**). hMSC treatment reduces glomerular hypertrophy on day 7 (**c, d**) and day 13 (**g, h**). hMSC treatment also reduces crescent formation on day 13 (**g, h**). Original magnifications, x200 (a, c, e, g), x1000 (b, d, f, h).

It appeared that the hMSCs, which were administered to rats 4 days after the induction of nephritis when the glomeruli showed prominent cellular crescents with fibrinoid necrosis, had a potential to improve renal histological findings. **Figs.2a and 2b** show typical silver-stained sections on day 7 in the WKY-HBSS rats. The glomeruli on day 7 showed severe fibrinoid necrosis and cellular crescent formation (**Fig.2a, b**). The crescentic glomeruli started to transform from cellular to fibrocellular, and there was some segmental glomerulosclerosis on day 13 (**Fig.2e, f**). The degree of crescentic glomerulonephritis was evaluated by measuring glomerular tuft area and by scoring crescent and fibrinoid necrosis in glomeruli. The quantitative analysis of the glomerular tuft area is presented in **Table 1**. Consistent with the urinary protein and kidney weight, hMSC treatment significantly reduced glomerular hypertrophy in rats with nephritis on day 7 (P<0.05) and day 13 (P<0.05) compared to HBSS treatment (**Fig.2c, d** **, and** **Table 1** **)**. hMSC treatment also significantly reduced crescent formation on day 13 (P<0.05) (**Fig.2g, h** **, and** **Table 1** **)**. However, fibrinoid necrosis in glomeruli was identical between the HBSS-treated rats and MSC-treated rats (**Table 1** **)**.

**Table 1:** Assessment of histological findings.

	Day 7^a^		Day 13^b^	
Group	HBSS-WKY, n = 14	MSC-WKY, n = 11	HBSS-WKY, n = 10	MSC-WKY, n = 10
**Glomerular tuft area (µm^2^)**	8709.13±175.74	8322±94.49*	9627.10±473.2	8631.56±98.97*
**%Crescent**	90.27±0.78	89.00±1.14	93.00±1.27	93.11±1.01
**Crescent score (0**–**4)**	2.04±0.05	2.00±0.08	2.23±0.09	1.96±0.07*
**%Necrosis**	55.33±1.83	50.83±2.83	52.6±2.94	54.00±1.61

Data are means ± SEM.

Mann-Whitney test: *P<0.05 vs. WKY rats with the same duration of HBSS treatment.

a The rats were treated with either HBSS or MSCs at day 4, and sacrificed at day 7.

b The rats were treated with either HBSS or MSCs at day 4, and sacrificed at day 13.

### Results of the immunofluorescence studies

Prominent glomerular fibrin deposition was observed in the HBSS-treated rats on days 7 and 13. The semiquantitative evaluation of fibrin deposition showed that the fibrin scores were identical in the WKY-MSC rats and WKY-HBSS rats at each time point, which is consistent with the result for fibrinoid necrosis (fibrin score, Day 7: 2.01±0.03 *vs.* 1.92±0.07, NS; Day 13: 2.14±0.05 *vs.* 2.16±0.09, NS, WKY-HBSS rats *vs*. WKY-MSC rats). Rabbit IgG was detected in an intense linear pattern along the glomerular capillaries in the WKY-MSC rats and WKY-HBSS rats. Rat IgG was also detected along the glomerular capillaries. Rat C3 staining was faint at each time point. There was no significant difference in rabbit IgG, rat IgG, and rat C3 glomerular staining between the WKY-MSC rats and WKY-HBSS rats (data not shown). Furthermore, there was no significant difference in the levels of serum anti-rabbit IgG antibody between the WKY-MSC rats and WKY-HBSS rats (data not shown). These findings suggest that MSCs do not significantly affect the process of heterologous antibody deposition or autologous antibody production.

### Effects of hMSCs on apoptosis, CD8+ cell influx, and macrophage accumulation in rats with nephritis

**Figure 3:**
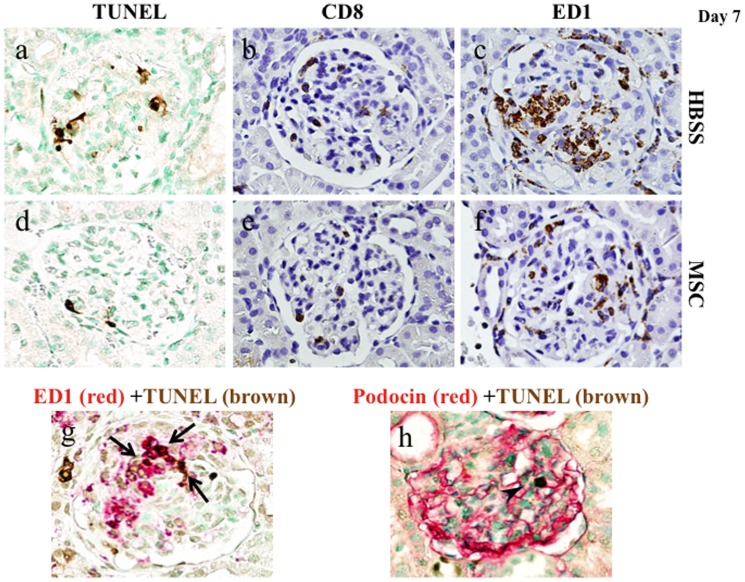
Immunohistochemistry for TUNEL, CD8, and ED-1 in the study groups. Representative pictures stained with immunohistochemistry for TUNEL (a, d), CD8 (b, e), and ED-1 (c, f) of an HBSS-treated rat (a–c) and an MSC-treated rat (d–f) on day 7. Kidney sections were stained using two-color immunohistochemistry with ED1 or podocin stained red and TUNEL stained brown in an HBSS-treated rat (g,h). Some portion of the TUNEL+ cells is stained with ED1+ (arrows) and podocin + (arrowhead), Original magnifications, x1000.

hMSCs significantly reduced apoptosis in rats with nephritis on both days 7 and 13, evaluated by counting the TUNEL+ cell number per glomerular cross-section (**Fig.3a, d** **, and** **Table 2**). Although some portion of the TUNEL+ cells was stained with ED1 or podocin (**Fig.3g,h** **)**, there was a significant number of TUNEL+ cells that were negative for ED1 or podocin. To identify the cell phenotype of leukocytes affected by MSCs, CD8+ and ED1+ cells in glomeruli were examined. The CD8+ cell number per glomerular cross-section on day 7 was less in the WKY-MSC rats than in the WKY-HBSS rats (**Fig.3b, e** **, and** **Table 2**). Immunostaining for ED1 in WKY-HBSS rats showed intense glomerular staining, whereas significantly less staining was observed in the WKY-MSC rats on day 7 (**Fig.3c, f** **, and** **Table 2**). The CD8+ cell number and immunostaining for ED1 on day 13 were identical between the WKY-MSC rats and the WKY-HBSS rats (**Table 2**).

**Table 2:** Results of immunohistochemistry.

	Day 7^a^		Day 13^b^	
Group	HBSS-WKY, n = 14	MSC-WKY, n = 11	HBSS-WKY, n = 10	MSC-WKY, n = 10
**TUNEL+ cells/glomerular cross-section**	1.62±0.12	1.13±0.06****	1.29±0.16	0.81±0.12*
**CD8+ cells/glomerular cross-section**	2.25±0.09	1.97±0.14*	2.29±0.15	2.03± 0.15
**ED1 score (0**–**3)**	1.81±0.05	1.67±0.04*	1.71±0.08	1.61±0.07

Data are means ± SEM.

Mann-Whitney test: *P<0.05, ****P<0.0001 vs. WKY rats with the same duration of HBSS treatment.

a The rats were treated with either HBSS or MSC at day 4, and sacrificed at day 7.

b The rats were treated with either HBSS or MSC at day 4, and sacrificed at day 13.

Semiquantitative assessment of the ED1 score and quantitative assessment of TUNEL+ and CD8+ cells per glomerular cross-section in each group. Each group contained 10–14 rats, and 50 glomeruli per rat were evaluated in a blind fashion.

### Effects of hMSCs on renal cortical proinflammatory cytokine expression in rats with nephritis

Since hMSCs significantly reduced inflammatory cell infiltration in glomeruli on day 7, proinflammatory cytokines, which are fundamental in the pathogenesis of crescentic glomerulonephritis, were examined by real-time RT-PCR. The gene expression levels of TNF-α, IL-1β, MCP-1, and IL-6, which are known as proinflammatory cytokines, were much higher in the WKY-HBSS rats than in WKY rats without nephritis. hMSCs significantly decreased gene expression levels of TNF-α (P<0.0001) and IL-1β(P<0.001) but not MCP-1 or IL-6 on day 7 (**Table 3**).

**Table 3:** Results of real-time RT-PCR for cytokine genes in renal cortex.

	Day7^a^		Day13^b^	
Group	HBSS-WKY	MSC-WKY	HBSS-WKY	MSC-WKY
**TNF-α**	11.31±0.24	6.00±0.43****	7.36±0.85	6.37±0.57
**IL-1β**	16.40±1.58	11.72±0.86***	13.02±1.17	14.70±1.23
**MCP-1**	154.63±13.68	130.91±10.45	133.86±11.24	136.95±8.02
**IL-6**	90.56±10.32	78.26±7.76	78.68±7.36	74.98±3.67
**IFN-γ**	50.52±7.49	51.05±5.61	35.03±2.31	38.56±4.37
**IL-4**	3.54±0.48	7.27±2.16*	4.11±0.51	3.16±0.06
**IL-10**	5.50±0.38	5.48±0.35	5.30±0.57	5.71±0.58
**IL-17**	12.18±1.34	9.14±0.97**	12.85±1.72	11.31±1.36
**FOXP3**	10.97±0.86	15.44±1.69**	13.55±2.31	10.63±1.02

Real-time RT-PCR for cytokines and profibrogenic genes in each group. Data are mean ± SEM.

a The rats were treated with either HBSS or MSC at day 4, and sacrificed at day 7.

b The rats were treated with either HBSS or MSC at day 4, and sacrificed at day 13.

Mann-Whitney test: *P<0.05, **P<0.01, ***P<0.001, ****P<0.0001 *vs.* WKY rats with same duration of HBSS treatment.

The values were normalized to the GAPDH values and then expressed as relative quantification.

**Figure 4:**
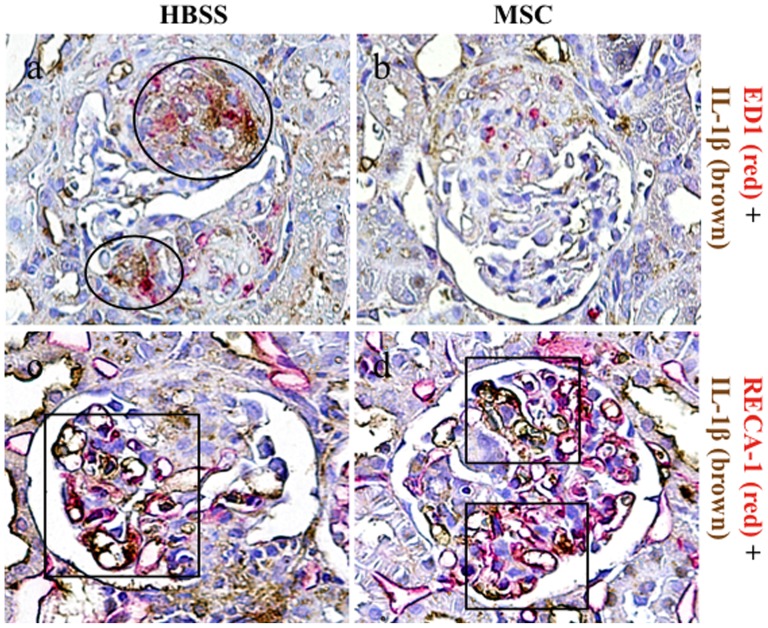
Double immunostaining for ED1 or RECA-1 with IL-1β in the study groups. Kidney sections were stained using two-color immunohistochemistry with ED1 or RECA-1 stained red and IL-1β stained brown in an HBSS-treated rat with nephritis (**a,c**) and an MSC-treated rat with nephritis (**b,d**). A large number of ED1+ macrophages shows double staining for IL-1β in the WKY-HBSS rats (circles) (**a**). RECA-1 is partially double-stained with IL-1β in both the WKY-HBSS rats and the WKY-MSC rats (squares) (**c,d**). Original magnifications, x1000.

Double immunostaining of ED-1 with IL-1β was performed to confirm that a significant reduction of proinflammatory cytokines in glomeruli was associated with inhibition of macrophage accumulation by hMSCs (**Fig.4**). A large number of ED1+ macrophages exhibited double staining for IL-1β in the WKY-HBSS rats (**Fig.4a**). In the WKY-MSC rats, there was a reduction of glomerular macrophage accumulation, along with an inhibition of IL-1β expression that was co-localized with glomerular macrophages (**Fig.4b**). The endothelial cells are also known to be a local source of IL-1β. RECA-1, an endothelial cell surface marker, was partially double-stained with IL-1β in both the WKY-HBSS rats and the WKY-MSC rats (**Fig.4c,d**).

The proinflammatory cytokines were identical in the two groups on day 13 (**Table 3**).

### Effects of hMSCs on polarization of Th1/Th2 and Th17/Tregs in rats with nephritis

**Figure 5:**
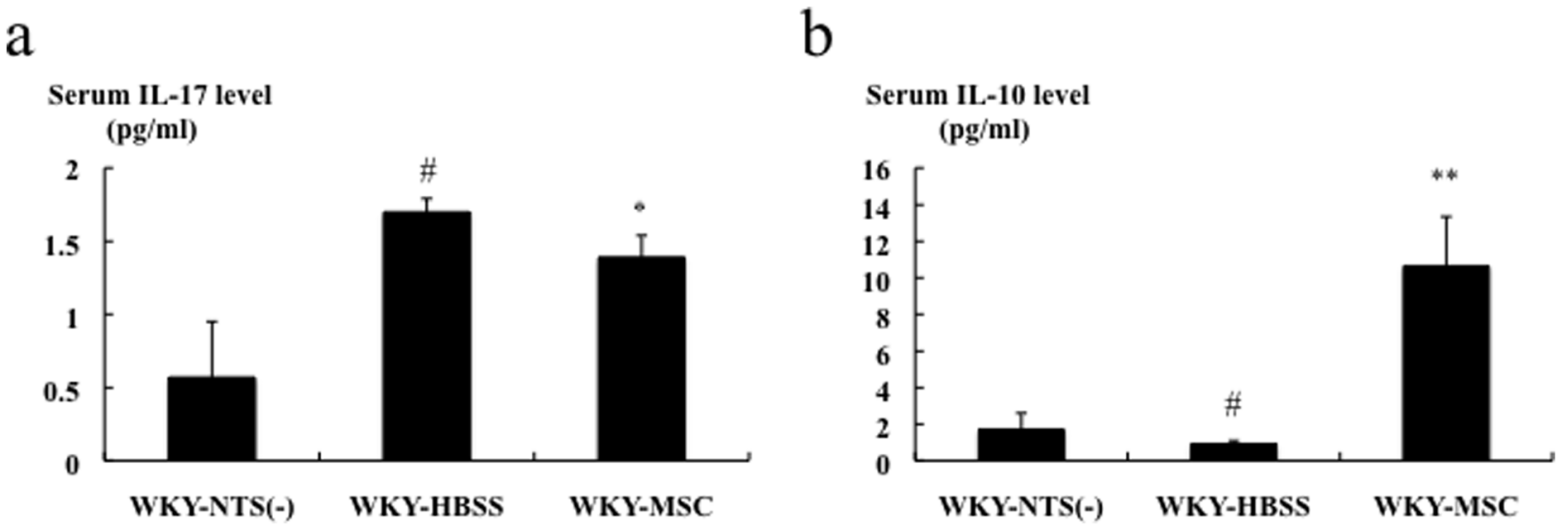
Effects of hMSCs on serum levels of IL-17 and IL-10 in rats with nephritis on day 7. Serum levels of IL-17 and IL-10 in rats with nephritis on day 7 measured by ELISA. Data are expressed as means ± SEM. Mann-Whitney test: *p<0.05, **p<0.01: HBSS-treated rats with nephritis vs. MSC-treated rats with nephritis; #p<0.05: HBSS-treated rats with nephritis vs. WKY rats without nephritis.

As shown in **Table 3**, renal cortical IFN-γ, IL-4, IL-10, IL-17, and Foxp3 gene expressions were significantly higher in the WKY-HBSS rats than in WKY rats without nephritis, as assessed by real-time RT-PCR. On day 7, IL-4 (P<0.05) and Foxp3 (P<0.01) gene expressions were significantly upregulated, whereas IL-17 (P<0.01) gene expression was significantly downregulated by hMSC treatment. hMSC treatment did not affect IFN-γ and IL-10 gene expressions. On the other hand, these cytokines were identical in the two groups on day 13 (**Table 3**). For examination of the systemic effect of hMSCs on Th17, Th1, and Th2, serum levels of IL-17, IFN-γ, and IL-10 were measured in HBSS-treated rats and MSC-treated rats on day 7 using ELISA kits. As shown in **Figure 5**, hMSC treatment was associated with a significant decrease in the serum IL-17 level and a significant increase in the serum IL-10 level in rats with nephritis. The serum IFN-γ level in the majority of the samples of the study groups was below the detection level (data not shown).

Next, the population of Tregs in the spleen was compared between the study groups on day 7 using flow cytometry. There was no significant difference in the population of CD4+CD25+FoxP3+ cells in the spleen between the study groups (4.64%±0.11% *vs.* 4.82%±0.15% *vs.* 4.43%±0.05%, NS; WKY-HBSS rats *vs*. WKY-MSC rats *vs.* WKY rats without nephritis).

Furthermore, serum IL-1β and IL-6 levels were measured using ELISA kits to evaluate if the prominent IL-10 increase induced systemic reset of the inflammatory phenotype. The serum IL-1β level in the majority of the samples of the study groups was below the detection level (data not shown), whereas the serum IL-6 level was identical in the HBSS-treated rats and MSC-treated rats on day 7 (116.62±1.34 pg/mL *vs.* 115.08±3.68 pg/mL; WKY-HBSS rats *vs*. WKY-MSC rats).

On the other hand, the effects of MSCs on podocytes, endothelium, and mesangium on day 7 were analyzed by measuring gene expression levels of nephrin, PAI-1, and PDGF-B by real-time RT-PCR. PAI-1 gene expression was significantly downregulated by hMSC treatment, whereas hMSC treatment did not affect nephrin and PDGF-B gene expressions significantly (Nephrin: 0.13±0.02 *vs.* 0.16±0.02, NS; PAI-1: 47.36±4.54 *vs.* 35.94±2.80, P<0.05; PDGF-B: 2.83±0.14 *vs.* 2.90±0.19, NS; WKY-HBSS rats *vs*. WKY-MSC rats).

### Effects of hMSCs on renal cortical profibrogenic gene expression and protein in rats with nephritis on day 13

**Figure 6:**
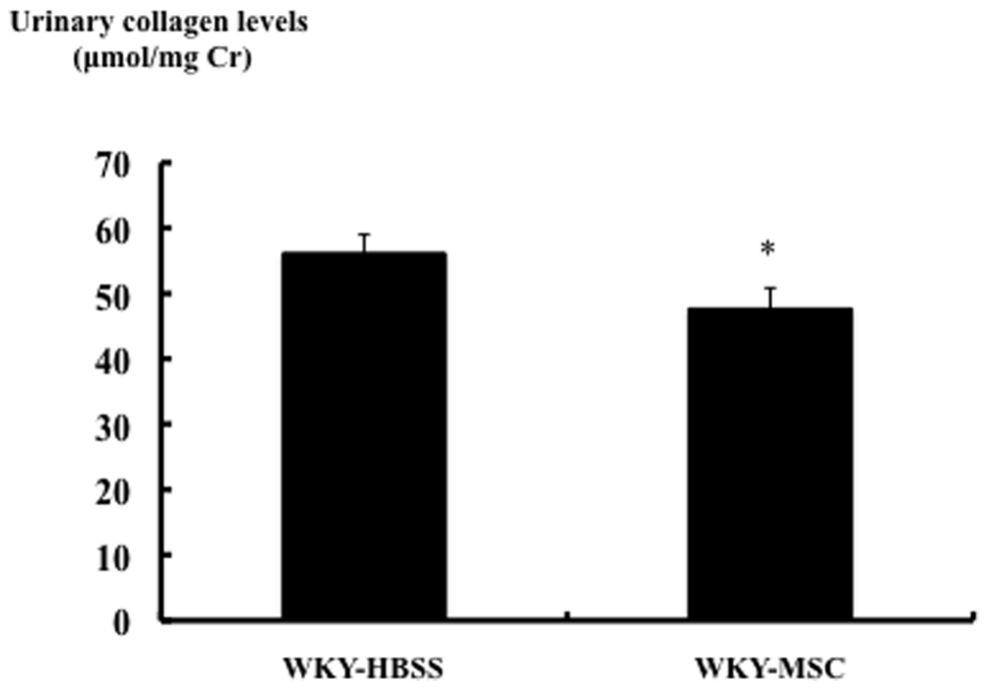
Effects of MSCs on urinary collagen levels in the study groups. Urinary collagen levels of each group measured as described in materials and methods. Data are expressed as means ± SEM. Mann-Whitney test: *p<0.05; HBSS-treated rats with nephritis vs. MSC-treated rats with nephritis.

The gene expression levels of collagen type I and type III were much higher in the WKY-HBSS rats than in WKY rats without nephritis, as assessed by real-time RT-PCR. hMSCs significantly decreased collagen type I and type III gene expressions in rats with nephritis. TGF-b gene expression was increased in the WKY-HBSS rats compared to WKY rats without nephritis. TGF-β gene expression in the kidneys of rats with nephritis was attenuated by hMSCs (**Table 4**). In order to verify the decrease in collagen and TGF-β gene expressions by hMSCs, urinary total collagen levels were determined by analysis of urinary hydroxyproline content. The urinary total collagen level was significantly less in the WKY-MSC rats than in the WKY-HBSS rats (**Fig. 6** **)**.

**Table 4:** Results of real-time RT-PCR for profibrogenic genes in renal cortex on day 13.

Group	HBSS-WKY, n = 10	MSC-WKY, n = 10
**Collagen type I**	24.37±4.42	13.62±1.20**
**Collagen type III**	29.29±2.31	21.23±1.58**
**TGF-β**	5.93±0.43	4.94±0.23*

Real-time RT-PCR for profibrogenic genes. Data are means±SEM.

Mann-Whitney test: *P<0.05, **P<0.01 *vs.* WKY rats with HBSS treatment.

The values were normalized to the GAPDH values and then expressed as relative quantification.

### Detection of CM-DiI-labeled hMSCs in kidney and other organs

**Figure 7:**
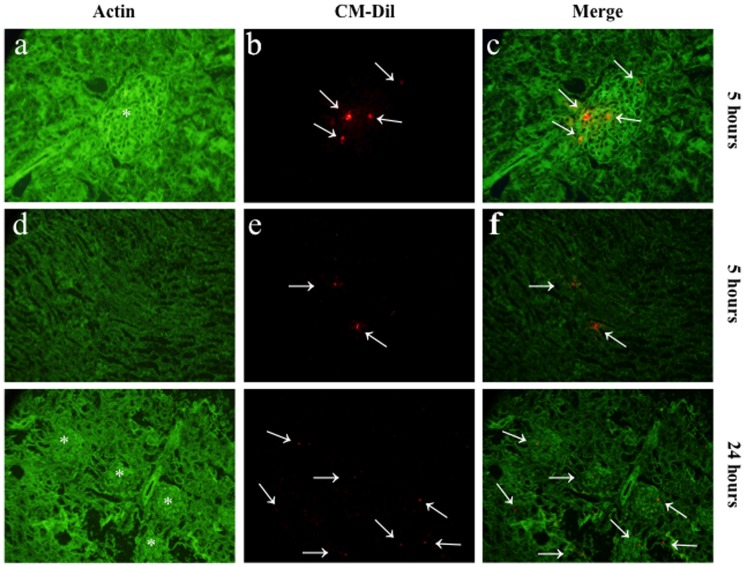
Detection of CM-DiI-labeled MSCs in kidneys. Representative pictures of frozen tissue sections of kidney of an MSC-treated rat at 5 h (a–f) or 24 h (g-i) after CM-DiI-labeled hMSC administration. The presence of CM-DiI-labeled hMSCs (red stained cells; arrows) was examined by counterstaining with actin antibody (green staining). Original magnifications, x400 (a–f), x200 (g–i). Asterisk (*) represents glomerulus.

**Figure 8:**
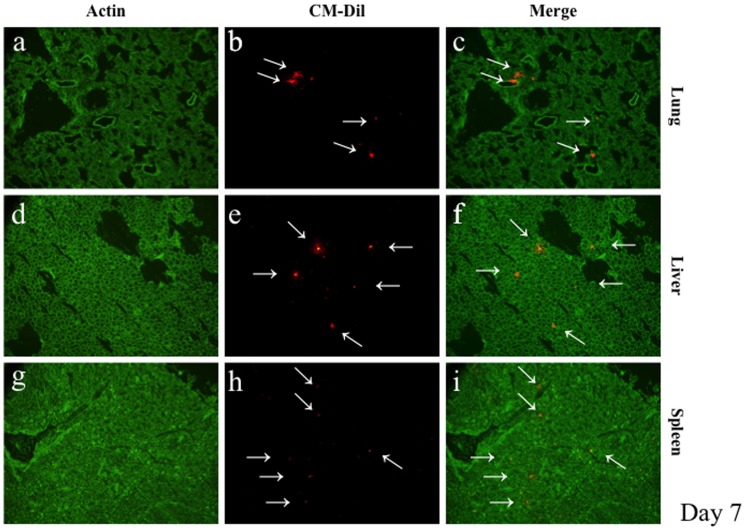
Detection of CM-DiI-labeled MSCs in lungs, liver, and spleen. Representative pictures of frozen tissue sections of lungs, liver, and spleen of an MSC-treated rat at 5 h after CM-DiI-labeled hMSC administration. The presence of CM-DiI-labeled hMSCs (red stained cells; arrows) was examined by counterstaining with actin antibody (green staining). Original magnifications, x200 (a–f).

Twelve rats with nephritis and two rats without nephritis were administered hMSCs that were fluorescently labeled using conjugated red fluorochrome CellTracker CM-DiI to evaluate the localization of hMSCs in kidney and other organs. The rats were sacrificed at 5 h, 24 h, 48 h, 7 days, or 13 days after CM-DiI-labeled hMSC administration. hMSCs were detected in the kidneys of the rat at 5 h and persisted until 48 h **(** **Fig. 7** **)**, but hMSCs were barely detectable at 7 days and 13 days (data not shown). From 5 h to 48 h, hMSCs were localized mostly in the glomeruli and slightly in the tubulointerstitium **(** **Fig. 7g**–**7i** **)**. Fluorescent-labeled hMSCs were also present in lung, heart, liver, and spleen **(** **Fig. 8** **).** The number of hMSCs in each organ was graded, as shown in **Table 5**.

## Discussion

The major findings in the present study were: 1) the administration of hMSCs, which migrate to glomeruli and the tubulointerstitium, demonstrated both a functional and histological benefit in rats with anti-GBM GN when given 4 days after the administration of nephrotoxic serum, as evaluated by a significant reduction of the urinary protein level, serum Cr level, kidney weight, glomerular tuft area, and degree of glomerular crescent formation; 2) there was a significant reduction in macrophage and CD8+ cell infiltration in the glomeruli, which are largely involved in the pathogenesis of this model of GN, concomitant with a significant reduction of proinflammatory cytokine mRNA in the renal cortex; 3) the beneficial effects of hMSCs in the early phase of anti-GBM GN appear to be mediated in part by modification of the Th1/Th2 and/or Th17/Tregs balance; renal cortical mRNA for IL-17 and the serum IL-17A level were decreased, whereas renal cortical mRNA for IL-4 and Foxp3 and the serum IL-10 level were increased in the MSC-treated group; and 4) MSC treatment prevented the development of glomerular fibrosis in anti-GBM GN, as evaluated by a significant reduction of collagen types I and III and TGF-β mRNA. This was reflected in the protein levels of total collagen in urine.

The most salient effect of MSCs on the biochemical parameters in rats with anti-GBM GN was the alleviation of proteinuria and reduction of kidney weight. Although MSCs did not affect the proportion of glomeruli that exhibited crescents and/or necrosis, MSCs significantly decreased glomerular tuft area and the degree of glomerular crescent formation. Furthermore, serum Cr levels started to decrease on day 13. The first explanation for the improvement of renal function despite the lack of a significant influence on crescent incidence and necrosis on day 13 was that MSCs attenuated the glomerular hypertension, as represented by decreased glomerular tuft area. The alternate explanation was that MSCs attenuated the severe prerenal factor with lower plasma volume and higher renin-angiotensin system (RAS) activation by reducing proteinuria. On the other hand, there is a possibility that the cellular regeneration in the endothelium by hMSCs contributed to the partial amelioration of proteinuria, because hMSCs significantly reduced cortical PAI-1 mRNA.

According to previous reports, macrophages [33]–[36] and CD8-positive cells [33], [37]–[39] have been shown to be crucial for the initiation and subsequent progression of anti-GBM nephritis in WKY rats. As indicated in these reports, ED1-positive macrophages and CD8-positive cells accumulated significantly in glomeruli in HBSS-treated rats with nephritis in the present study. It was found that hMSC administration reduced glomerular macrophage influx, which in turn suppressed mRNA expression of macrophage-associated proinflammatory cytokines, IL-1β and TNF-α in the renal cortex, which is considered an important inducer for chemokines in themselves or in combination with IL-17 in renal intrinsic cells [40], [41]. The double staining of ED-1-positive macrophages and IL-1β indicated that glomerular staining of IL-1β was macrophage-dependent, and the double staining was abolished, along with macrophage reduction, after hMSC treatment in nephritis. This effect was accompanied by reduction of proteinuria, kidney weight, and glomerular tuft area. Although little evidence is available concerning the potential mechanisms of macrophage reduction by hMSC treatment, hMSCs have been shown to inhibit the differentiation of monocytes into immature dendritic cells (DCs) [42] by blocking the monocyte cell cycle at the G0 phase [43]. In addition, hMSCs decrease TNF-a production in immature DCs [44], which is consistent with the present result that hMSCs significantly decreased TNF-αmRNA expression in renal cortex concomitant with glomerular macrophage reduction in nephritis.

On the other hand, MSC treatment significantly reduced glomerular CD8-positive cell numbers in rats with nephritis. The role of CD8-positive cells as effector cells in this model was strongly suggested by a previous report in which depletion of CD8-positive cells using anti-CD8 monoclonal antibody significantly prevented proteinuria and glomerular lesions [37]. These CD8-positive cells in the glomeruli were identified phenotypically as NK cells and not precytotoxic, cytotoxic, presuppressor, or suppressor cells, because of their lack of CD3, CD4, T cell receptor (TCR)-αβ or IL-2 receptor (α-chain) [37]. It has been reported that MSCs inhibit NK cell function along with downregulation of IFN-γ secretion [44] and NK cell proliferation stimulated by IL-2 [45]. These lines of evidence indicate that the decreased glomerular macrophage and CD8-positive cell accumulation with hMSC treatment could be explained by a direct effect of hMSCs, as well as the reduction of glomerular injury and the subsequent reduction of cytokines and chemotactic signals.

The reduction of TUNEL+ cell number in the MSC-treated group could be partially attributed to the inhibition of macrophages and the preservation of podocytes by hMSCs, because some TUNEL+ cells were positive for ED1+ macrophages or podocin+ podocytes. We presume that a large number of TUNEL+ cells that were negative for ED1 or podocin in the crescent area were parietal epithelial cells (PECs), which have recently been thought to be a main component of cellular crescents [46].

The Th1 response has been reported to play an important role in the pathogenesis of anti-GBM GN [47], [48]; however, recent studies have identified Th17 cells as key players in GN [18], [19]. Tregs, known as the functional antagonists of Th17 cells, are critical for the control of autoimmunity and tissue injury [49]. Therefore, modification of the Th1/Th2 and/or Th17/Tregs balance is one of the therapeutic options for this disease. The murine anti-GBM GN was reduced by administration of Th2 cytokines [20] and endogenous Tregs [21], and it was worse when GN was induced in Treg-depleted mice [22]. MSC treatment has been shown to suppress the Th1 and Th17 response and to induce the Th2 [50] and Treg response [51], [52], [53]. Ghannam et al. reported that MSCs mediated the adhesion of Th17 cells via CCR6 under inflammatory conditions and exert anti-inflammatory effects through the induction of Tregs [51]. Consistent with previous reports, the expression of Th17 cytokine IL-17 mRNA was inhibited by hMSCs, and this was concomitant with an increase in the production of Foxp3 mRNA, which is a transcription factor specifically expressed by Treg cells. We also found that hMSC treatment significantly reduced the serum IL-17A level. Taken together, this demonstrated that hMSCs could alter the systemic immune response in anti-GBM GN via interacting with Th1/Th2 and Th17/Treg cells in kidneys and/or other immune organs.

The discrepancy between MSC-associated high circulating IL-10 serum levels with no change in IL-10 mRNA expression in the cortex suggests that MSCs displayed extra-renal immunomodulatory actions. There is a large body of evidence that MSCs suppress inflammation and enhance tissue repair by modulating the phenotype of macrophages from an M1 phenotype to the alternatively activated or anti-inflammatory M2 phenotype [50], [54], [55]. Nemeth et al. showed that MSCs attenuated sepsis in mice, and the therapeutic effect was eliminated by macrophage depletion or by antibodies against IL-10 or IL-10 receptor [50]. The authors also showed that lipopolysaccharide (LPS)-stimulated macrophages produced more IL-10 when cultured with MSCs *in vitro*, and they demonstrated that this effect was due to an increased release of prostaglandin E2 (PGE2) by MSCs acting on the prostaglandin EP2 and EP4 receptors on macrophages. Moreover, it has been shown that co-culture with MSCs induced macrophages to adopt an enhanced regulatory phenotype via expression of increased levels of IL-10 and reduced levels of TNF-α and IL-12 that was mediated by PGE2, while showing higher phagocytic activity [54], [55]. These lines of evidence suggest that the therapeutic action mediated by MSCs in anti-GBM GN was also induced in part through the stimulation of IL-10 production by macrophages.

The present observations that the number and density of MSCs in the kidney structures appeared relatively low, which indicates that most of the 3 million injected cells were trapped outside the kidney, including the lungs, in which MSCs were the most numerous in the present study, also suggest the paracrine effect of MSCs. Recent evidence showed that the infused hMSCs in mice were trapped in the lung as micro-emboli, and the cells were activated by signals from the injured heart to synthesize and secrete the anti-inflammatory protein TSG-6 [15].

The present results demonstrated that anti-inflammatory and immunomodulatory effects were involved in the mechanism of attenuating established experimental anti-GBM GN by hMSCs. These results suggest that hMSCs are a promising therapeutic candidate for the treatment of anti-GBM GN.

## Acknowledgments

The authors would like to thank Ms. Tomoko Suzuki, Ms. Naoko Ono, Sayuko Kasahara, and Ms. Fumiko Kondo for their excellent technical assistance.
